# Poly[[bis­[μ_4_-3-(2-carboxyl­atophen­yl)propionato]{*N*-[2-(pyridine-3-amido)­eth­yl]nicotinamide}­dicopper(II)] penta­hydrate]

**DOI:** 10.1107/S2414314623006223

**Published:** 2023-07-22

**Authors:** Gabrielle J. Gaskin, Robert L. LaDuca

**Affiliations:** a E-35 Holmes Hall, Michigan State University, Lyman Briggs College, 919 E. Shaw Lane, East Lansing, MI 48825, USA; Purdue University, USA

**Keywords:** crystal structure, copper(II), self-penetration, coordination polymer

## Abstract

In the title compound, {[Cu_2_(C_10_H_8_O_4_)_2_(C_14_H_14_N_4_O_2_)]·5H_2_O}_*n*_, the Cu^II^ cations are coordinated in a square-pyramidal fashion, with four 3-(2-carb­oxy­phen­yl)propionate (cpp) carboxyl­ate O-atom donors in the basal plane, along with an N-atom donor from a *N*-[2-(pyridin-3-yl­amino)­eth­yl]nicotinamide (pen) ligand in the apical position. [Cu_2_(cpp)_2_]_*n*_ coordination polymer layer motifs with embedded {Cu_2_(OCO)_4_} paddlewheel clusters are thereby constructed. These layer motifs are connected into a three-dimensional [Cu_2_(cpp)_2_(pen)]_*n*_ coordination polymer network by tethering pen ligands. Treating the {Cu_2_(OCO)_4_ paddelwheel clusters as 6-connected nodes reveals an underlying cross-pillared self-penetrated **rob** network with 4^8^6^6^8 topology. The water mol­ecules of crystallization are held to the coordination polymer network by N—H⋯O and O—H⋯O hydrogen-bonding patterns.

## Abstract 2

A divalent copper three-dimensional self-penetrated coordination polymer with **rob** topology, {[Cu_2_(3-(2-carb­oxy­phen­yl)propionate)_2_(ethyl­enedi­amine­(bis­(nicotinamide)] ^.^5H_2_O}_*n*_, was structurally characterized by single-crystal X-ray diffraction.

## Graphical abstract

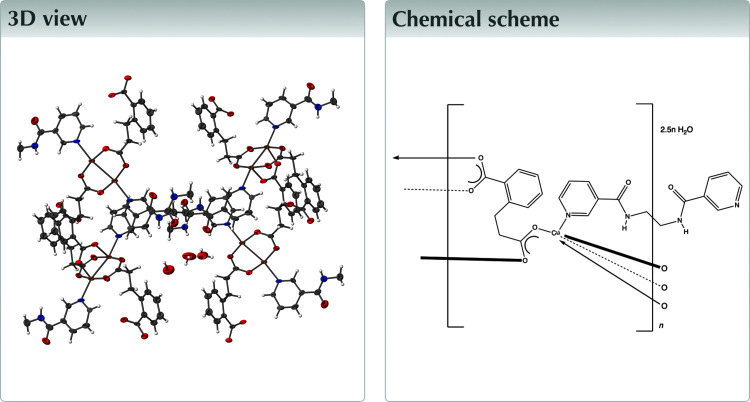

## Structure description

The title compound was isolated during an exploratory synthetic effort aiming to produce a copper coordination polymer containing both 3-(2-carb­oxy­phen­yl)propionic (cpp) and *N*-(2-(pyridin-3-yl­amino)­eth­yl)nicotinamide (pen) ligands. The pen ligand has to date seldom been used in coordination polymer chemistry (Wang *et al.*, 2013 ▸). Our group has previously reported a racemic cobalt camphorate pen-containing coordination polymer with 4^12^6^3^ **pcu** topology (Przybyla *et al.*, 2019 ▸)

**Figure 1:**
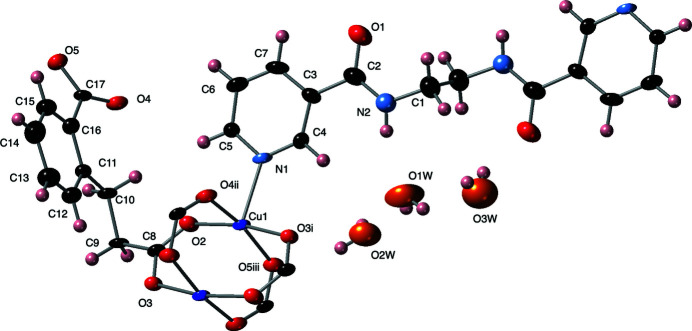
Coordination environment in the title compound with full ligand set and complete {Cu_2_(OCO)_4_} paddlewheel cluster. Water mol­ecules of crystallization are shown. Displacement ellipsoids are drawn at the 50% probability level. Color code: Cu, dark blue; O in ligands, red; O in water mol­ecules of crystallization, orange; N, light blue; C, black; H, pink. Symmetry codes are as listed in Table 1 ▸.

The asymmetric unit of the title compound contains a divalent copper atom, a fully deprotonated cpp ligand, half of a pen ligand whose central ethyl­ene moiety is sited over a crystallographic inversion center (Wyckoff special position *a*), two water mol­ecules of crystallization located on general positions, and one water mol­ecule of crystallization best refined at half occupancy and disordered about a crystallographic twofold rotation axis (Wyckoff special position *e*). The copper atoms in the title compound display a {CuNO_4_} square-pyramidal coordination environment (Fig. 1 ▸), with a pyridyl nitro­gen donor atom from a pen ligand located in the elongated apical position. Carboxyl­ate oxygen atom donors from four different cpp ligands occupy the basal plane, with the two ‘longer-arm’ ethyl-carboxyl­ate group oxygen atoms in *trans* position to each other, and the two ‘shorter-arm’ benzoate carboxyl­ate group oxygen atoms in the other two positions, also *trans* to each other. Bond lengths and angles within the coordination environment in the title compound are listed in Table 1 ▸.

**Figure 2:**
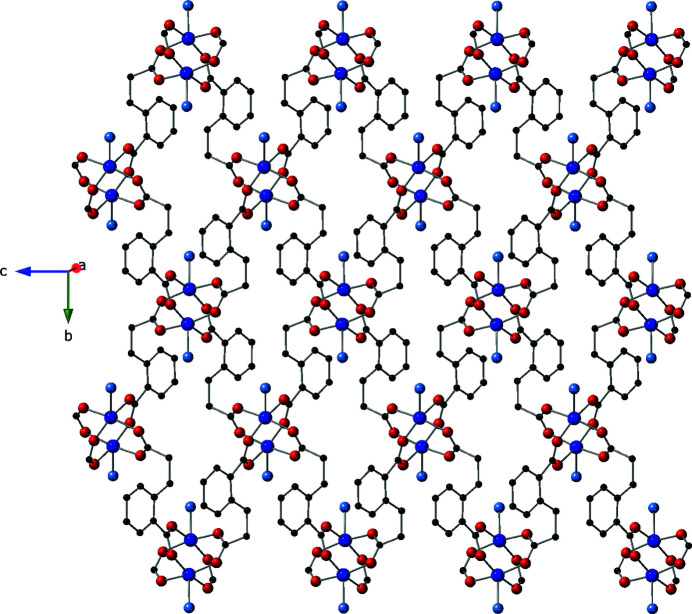
[Cu_2_(cpp)_2_]_*n*_ coordination polymer layer in the title compound, featuring {Cu_2_(OCO)_4_} paddlewheel clusters. Water mol­ecules of crystallization have been omitted.

**Figure 3:**
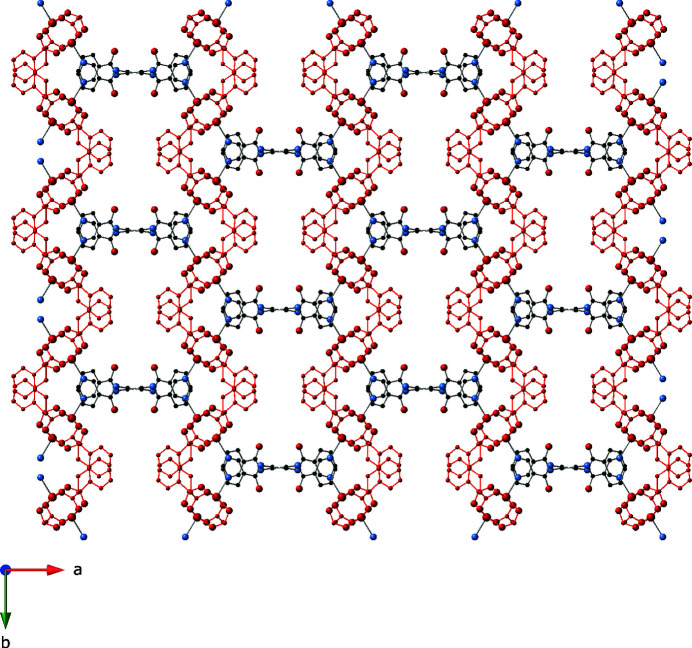
[Cu_2_(cpp)_2_(pen)]_*n*_ coordination polymer network in the title compound, with [Cu_2_(cpp)_2_]_*n*_ layers drawn in red

**Figure 4:**
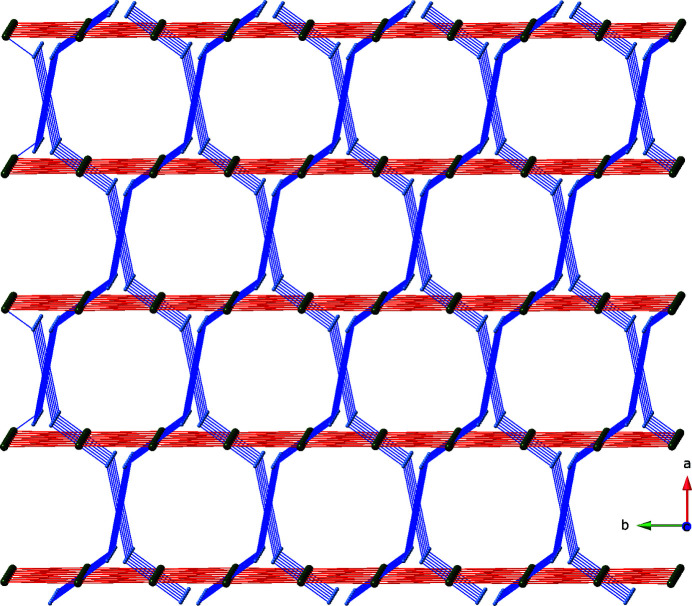
Schematic perspective of the 4^8^6^6^8 **rob** cross-pillared self-penetrated topology in the title compound. The spheres represent the centroids of the 6-connected {Cu_2_(OCO)_4_} paddlewheel clusters. The [Cu_2_(cpp)_2_]_*n*_ layers are drawn in red, with the cross-pillaring pen ligands drawn in blue.

The cpp ligands in the title compound connect four copper atoms in an exo­tetra­dentate pattern in which each carboxyl­ate oxygen atom binds only to one copper atom. Via four cpp carboxyl­ate groups – two from the longer-arm cpp termini and two from the shorter-arm cpp termini – {Cu_2_(OCO)_4_} paddlewheel dimeric units are formed (Fig. 1 ▸). These have a through-space Cu⋯Cu inter­nuclear distance of 2.620 (1) Å, and their centroids are located on crystallographic inversion centers (Wyckoff special position *d*). Each of these dimeric units connects to four others through the full span of the cpp ligands thereby forming [Cu_2_(cpp)_2_]_*n*_ coordination polymer layers arranged parallel to the *bc* crystal planes (Fig. 2 ▸). Parallel [Cu_2_(cpp)_2_]_*n*_ coordination polymer layers are pillared into a three-dimensional [Cu_2_(cpp)_2_(pen)]_*n*_ coordination polymer network (Fig. 3 ▸) by *anti*-conformation ebn ligands that span a Cu⋯Cu distance of 14.87 (1) Å. The nearest inter­nuclear distance between adjacent [Cu_2_(cpp)_2_]_*n*_ layers is 12.08 (1) Å, which is too short to be spanned directly by the pen ligands given their conformational constraints. As a result, the three-dimensional [Cu_2_(cpp)_2_(pen)]_*n*_ network is formed by cross-pillaring of the pen tethers, thereby precluding a much more common straight-pillared 4^12^6^3^ **pcu** topology. Treating each {Cu_2_(OCO)_4_} paddlewheel dimeric unit as a 6-connected node results in an uncommon self-penetrated, cross-pillared **rob** network with 4^8^6^6^8 topology when analyzed using the *TOPOS* software (Blatov *et al.*, 2014 ▸) (Fig. 4 ▸). Discrete D(3) short water mol­ecule chains (Infantes & Motherwell, 2002 ▸) positioned along the *c*-axis crystal direction occupy incipient channels comprising 21.8% of the unit cell volume according to *PLATON* (Spek, 2020 ▸).

The co-crystallized water mol­ecules are held to the coordination polymer framework by hydrogen-bonding acceptance from the pen N—H groups, and hydrogen-bonding donation to each other. Details regarding the hydrogen bonding in the title compound are listed in Table 2 ▸.

## Synthesis and crystallization

Cu(NO_3_)_2_^.^2.5 H_2_O (87 mg, 0.37 mmol), 3-(2-carb­oxy­phen­yl)propionic acid (cppH_2_) (73 mg, 0.37 mmol), *N*-(2-(pyridin-3-yl­amino)­eth­yl)nicotinamide (pen) (100 mg, 0.37 mmol) and 0.75 ml of a 1.0 *M* NaOH solution were placed into 10 ml distilled H_2_O in a Teflon-lined acid digestion bomb. The bomb was sealed and heated in an oven at 373 K for 48 h, and then cooled slowly to 273 K. Green crystals of the title complex were obtained in 43% yield.

## Refinement

Crystal data, data collection and structure refinement details are summarized in Table 3 ▸. The O3*W* position disordered across an inversion center was treated with a PART −1 command. Water H atom positions are refined, but suitably restrained with 0.84 (2) Å target values for O—H bonds, 1.36 (2) Å target values for H⋯H distances, and suitable *DFIX* commands for hydrogen-bonding H⋯O distances (with EQIV commands added as needed). The amine H-atom position was allowed to refine with an N—H distance restraint of 0.88 (2) Å.

## Supplementary Material

Crystal structure: contains datablock(s) I. DOI: 10.1107/S2414314623006223/zl4055sup1.cif

Structure factors: contains datablock(s) I. DOI: 10.1107/S2414314623006223/zl4055Isup3.hkl

CCDC reference: 1979279

Additional supporting information:  crystallographic information; 3D view; checkCIF report

## Figures and Tables

**Table 1:** Selected geometric parameters (Å, °)

Cu1—O2	1.965 (5)	Cu1—O5^iii^	1.975 (5)
Cu1—O3^i^	1.967 (5)	Cu1—N1	2.172 (6)
Cu1—O4^ii^	1.991 (5)		
			
O2—Cu1—O3^i^	168.9 (2)	O3^i^—Cu1—O5^iii^	87.1 (2)
O2—Cu1—O4^ii^	90.1 (2)	O3^i^—Cu1—N1	97.5 (2)
O2—Cu1—O5^iii^	91.5 (2)	O4^ii^—Cu1—N1	87.0 (2)
O2—Cu1—N1	93.6 (2)	O5^iii^—Cu1—O4^ii^	168.0 (2)
O3^i^—Cu1—O4^ii^	89.0 (2)	O5^iii^—Cu1—N1	104.8 (2)

Symmetry codes: (i) 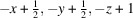 ; (ii) 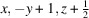 ; (iii) 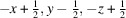 .

**Table 2:** Hydrogen-bond geometry (Å, °)

*D*—H⋯*A*	*D*—H	H⋯*A*	*D*⋯*A*	*D*—H⋯*A*
N2—H2⋯O1*W*	0.87 (2)	2.03 (4)	2.844 (10)	155 (8)
C5—H5⋯O2	0.95	2.50	3.099 (9)	121
C6—H6⋯O3^iv^	0.95	2.48	3.332 (9)	149
O1*W*—H1*WA*⋯O2*W*	0.85 (2)	2.00 (11)	2.698 (13)	139 (15)
O1*W*—H1*WB*⋯O3*W*	0.85 (2)	2.10 (2)	2.79 (2)	139 (5)
O2*W*—H2*WA*⋯O5^iii^	0.85 (2)	2.09 (2)	2.909 (10)	162 (7)
O2*W*—H2*WB*⋯O1^v^	0.85 (2)	2.06 (2)	2.816 (9)	147 (5)
O3*W*—H3*WA*⋯O1^ii^	0.84 (2)	2.10 (2)	2.917 (19)	162 (9)
O3*W*—H3*WB*⋯O1^vi^	0.84 (2)	2.10 (2)	2.848 (19)	147 (6)

Symmetry codes: (ii) 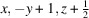 ; (iii) 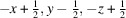 ; (iv) 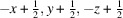 ; (v) 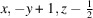 ; (vi) 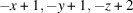 .

**Table 3:** Experimental details

Crystal data	
Chemical formula	[Cu_2_(C_10_H_8_O_4_)_2_(C_14_H_14_N_4_O_2_)]·5H_2_O
*M*_r_	871.78
Crystal system, space group	Monoclinic, *C*2/*c*
Temperature (K)	173
*a*, *b*, *c* (Å)	27.558 (10), 14.873 (5), 9.146 (3)
β (°)	98.396 (4)
*V* (Å^3^)	3709 (2)
*Z*	4
Radiation type	Mo *K*α
μ (mm^−1^)	1.22
Crystal size (mm)	0.28 × 0.16 × 0.11

Data collection	
Diffractometer	Bruker APEXII CCD
Absorption correction	Multi-scan (*SADABS*; Krause *et al.*, 2015 ▸)
*T*_min_, *T*_max_	0.560, 0.745
No. of measured, independent and observed [*I* > 2σ(*I*)] reflections	14392, 3393, 2011
*R*_int_	0.120
(sin θ/λ)_max_ (Å^−1^)	0.604

Refinement	
*R*[*F* ^2^ > 2σ(*F* ^2^)], *wR*(*F* ^2^), *S*	0.079, 0.230, 1.02
No. of reflections	3393
No. of parameters	274
No. of restraints	15
H-atom treatment	H atoms treated by a mixture of independent and constrained refinement
Δρ_max_, Δρ_min_ (e Å^−3^)	1.68, −0.61

Computer programs: *COSMO* (Bruker, 2009 ▸), *SAINT* (Bruker, 2014 ▸), *SHELXT2018/2* (Sheldrick, 2015 ▸), *SHELXL2018/3* (Sheldrick, 2015 ▸), *CrystalMaker X* (Palmer, 2020 ▸), and *OLEX2* (Dolomanov *et al.*, 2009 ▸).

## full crystallographic data

### Crystal data

**Table:**

[Cu_2_(C_10_H_8_O_4_)_2_(C_14_H_14_N_4_O_2_)]·5H_2_O	*F*(000) = 1800
*M**_r_* = 871.78	*D*_x_ = 1.561 Mg m^−^^3^
Monoclinic, *C*2/*c*	Mo *K*α radiation, λ = 0.71073 Å
*a* = 27.558 (10) Å	Cell parameters from 2345 reflections
*b* = 14.873 (5) Å	θ = 2.6–24.4°
*c* = 9.146 (3) Å	µ = 1.22 mm^−^^1^
β = 98.396 (4)°	*T* = 173 K
*V* = 3709 (2) Å^3^	Block, green
*Z* = 4	0.28 × 0.16 × 0.11 mm

### Data collection

**Table:**

Bruker APEXII CCD diffractometer	2011 reflections with *I* > 2σ(*I*)
φ and ω scans	*R*_int_ = 0.120
Absorption correction: multi-scan (SADABS; Krause *et al.*, 2015)	θ_max_ = 25.4°, θ_min_ = 1.5°
*T*_min_ = 0.560, *T*_max_ = 0.745	*h* = −33→33
14392 measured reflections	*k* = −17→17
3393 independent reflections	*l* = −11→11

### Refinement

**Table:**

Refinement on *F*^2^	15 restraints
Least-squares matrix: full	Hydrogen site location: mixed
*R*[*F*^2^ > 2σ(*F*^2^)] = 0.079	H atoms treated by a mixture of independent and constrained refinement
*wR*(*F*^2^) = 0.230	*w* = 1/[σ^2^(*F*_o_^2^) + (0.1316*P*)^2^] where *P* = (*F*_o_^2^ + 2*F*_c_^2^)/3
*S* = 1.02	(Δ/σ)_max_ < 0.001
3393 reflections	Δρ_max_ = 1.68 e Å^−^^3^
274 parameters	Δρ_min_ = −0.61 e Å^−^^3^

### Special details

**Table:**

Geometry. All esds (except the esd in the dihedral angle between two l.s. planes) are estimated using the full covariance matrix. The cell esds are taken into account individually in the estimation of esds in distances, angles and torsion angles; correlations between esds in cell parameters are only used when they are defined by crystal symmetry. An approximate (isotropic) treatment of cell esds is used for estimating esds involving l.s. planes.

### Fractional atomic coordinates and isotropic or equivalent isotropic displacement parameters (Å^2^)

**Table:**

	*x*	*y*	*z*	*U*_iso_*/*U*_eq_	Occ. (<1)
Cu1	0.28340 (3)	0.31333 (5)	0.51554 (10)	0.0264 (3)	
O1	0.4310 (2)	0.6336 (4)	0.8536 (6)	0.0531 (16)	
O2	0.2511 (2)	0.3449 (3)	0.3163 (6)	0.0394 (14)	
O3	0.19296 (19)	0.2383 (3)	0.2891 (5)	0.0327 (13)	
O4	0.23078 (19)	0.6236 (3)	0.1031 (6)	0.0369 (13)	
O5	0.17254 (18)	0.7279 (3)	0.0656 (6)	0.0352 (13)	
N1	0.3245 (2)	0.4382 (4)	0.5434 (7)	0.0282 (14)	
N2	0.4359 (3)	0.4863 (5)	0.9037 (8)	0.0406 (17)	
H2	0.435 (3)	0.432 (3)	0.868 (9)	0.049*	
C1	0.4759 (3)	0.4959 (6)	1.0262 (9)	0.044 (2)	
H1A	0.476383	0.442866	1.091850	0.053*	
H1B	0.470134	0.549866	1.084528	0.053*	
C2	0.4170 (3)	0.5543 (5)	0.8263 (9)	0.037 (2)	
C3	0.3778 (3)	0.5378 (5)	0.7005 (8)	0.0319 (18)	
C4	0.3592 (3)	0.4538 (5)	0.6571 (8)	0.0281 (17)	
H4	0.372276	0.403411	0.713309	0.034*	
C5	0.3067 (2)	0.5096 (4)	0.4656 (7)	0.0219 (15)	
H5	0.281838	0.499813	0.383485	0.026*	
C6	0.3218 (3)	0.5968 (5)	0.4963 (9)	0.0368 (19)	
H6	0.308207	0.645667	0.437001	0.044*	
C7	0.3571 (3)	0.6102 (5)	0.6154 (9)	0.039 (2)	
H7	0.367833	0.669622	0.640970	0.047*	
C8	0.2140 (3)	0.3066 (5)	0.2454 (8)	0.0268 (16)	
C9	0.1944 (3)	0.3433 (4)	0.0944 (8)	0.0283 (17)	
H9A	0.160390	0.321624	0.065279	0.034*	
H9B	0.214539	0.320015	0.021644	0.034*	
C10	0.1947 (3)	0.4467 (4)	0.0900 (8)	0.0278 (17)	
H10A	0.227492	0.467709	0.135551	0.033*	
H10B	0.190022	0.465789	−0.014842	0.033*	
C11	0.1564 (3)	0.4942 (5)	0.1665 (7)	0.0254 (16)	
C12	0.1229 (3)	0.4454 (5)	0.2329 (8)	0.0333 (18)	
H12	0.125034	0.381659	0.232085	0.040*	
C13	0.0869 (3)	0.4842 (6)	0.2995 (10)	0.045 (2)	
H13	0.065171	0.448100	0.346293	0.054*	
C14	0.0824 (3)	0.5796 (6)	0.2977 (10)	0.045 (2)	
H14	0.056980	0.608167	0.340443	0.054*	
C15	0.1155 (3)	0.6296 (5)	0.2330 (8)	0.0349 (19)	
H15	0.112726	0.693235	0.230924	0.042*	
C16	0.1529 (3)	0.5884 (5)	0.1704 (8)	0.0299 (17)	
C17	0.1880 (3)	0.6497 (5)	0.1074 (8)	0.0286 (17)	
O1W	0.4372 (5)	0.2956 (5)	0.8813 (11)	0.120 (4)	
H1WA	0.449 (6)	0.275 (9)	0.807 (10)	0.181*	
H1WB	0.445 (5)	0.259 (4)	0.951 (7)	0.181*	
O2W	0.4262 (3)	0.2494 (5)	0.5930 (8)	0.088 (3)	
H2WA	0.3977 (19)	0.232 (7)	0.557 (11)	0.133*	
H2WB	0.438 (2)	0.273 (6)	0.522 (9)	0.133*	
O3W	0.4849 (7)	0.2581 (12)	1.166 (2)	0.111 (6)	0.5
H3WA	0.464 (4)	0.281 (12)	1.21 (2)	0.167*	0.5
H3WB	0.501 (2)	0.300 (9)	1.134 (16)	0.167*	0.5

### Atomic displacement parameters (Å^2^)

**Table:**

	*U*^11^	*U*^22^	*U*^33^	*U*^12^	*U*^13^	*U*^23^
Cu1	0.0419 (6)	0.0104 (5)	0.0247 (5)	−0.0009 (4)	−0.0023 (4)	0.0007 (4)
O1	0.063 (4)	0.049 (4)	0.043 (4)	−0.012 (3)	−0.006 (3)	−0.012 (3)
O2	0.052 (4)	0.026 (3)	0.036 (3)	−0.011 (3)	−0.011 (3)	0.006 (2)
O3	0.047 (3)	0.020 (3)	0.026 (3)	−0.005 (2)	−0.010 (2)	0.003 (2)
O4	0.040 (3)	0.017 (3)	0.055 (4)	−0.003 (2)	0.011 (3)	0.004 (2)
O5	0.042 (3)	0.022 (3)	0.041 (3)	0.001 (2)	0.005 (3)	0.008 (2)
N1	0.045 (4)	0.010 (3)	0.029 (3)	−0.001 (3)	0.005 (3)	0.001 (3)
N2	0.046 (4)	0.036 (4)	0.038 (4)	−0.004 (3)	0.000 (3)	0.003 (3)
C1	0.043 (5)	0.048 (6)	0.039 (5)	−0.004 (4)	0.000 (4)	0.001 (4)
C2	0.046 (5)	0.034 (5)	0.031 (5)	−0.013 (4)	0.009 (4)	0.001 (4)
C3	0.039 (5)	0.026 (4)	0.029 (4)	−0.011 (3)	−0.001 (3)	0.000 (3)
C4	0.037 (4)	0.024 (4)	0.021 (4)	0.002 (3)	−0.004 (3)	0.007 (3)
C5	0.025 (4)	0.022 (4)	0.020 (4)	−0.001 (3)	0.006 (3)	0.000 (3)
C6	0.049 (5)	0.019 (4)	0.043 (5)	0.000 (3)	0.009 (4)	0.006 (4)
C7	0.054 (5)	0.020 (4)	0.043 (5)	−0.009 (4)	0.006 (4)	−0.007 (4)
C8	0.041 (4)	0.015 (4)	0.024 (4)	0.001 (3)	0.000 (3)	0.001 (3)
C9	0.053 (5)	0.011 (3)	0.019 (4)	−0.001 (3)	−0.003 (3)	−0.004 (3)
C10	0.048 (5)	0.012 (4)	0.022 (4)	−0.002 (3)	0.001 (3)	0.000 (3)
C11	0.036 (4)	0.018 (4)	0.020 (4)	−0.003 (3)	−0.004 (3)	0.004 (3)
C12	0.046 (5)	0.024 (4)	0.029 (4)	−0.002 (3)	0.002 (4)	0.006 (3)
C13	0.051 (5)	0.035 (5)	0.048 (6)	−0.007 (4)	0.007 (4)	0.010 (4)
C14	0.039 (5)	0.050 (6)	0.048 (5)	0.004 (4)	0.011 (4)	0.001 (4)
C15	0.047 (5)	0.028 (4)	0.029 (4)	0.002 (4)	0.003 (4)	−0.005 (3)
C16	0.037 (4)	0.024 (4)	0.026 (4)	−0.002 (3)	−0.002 (3)	0.009 (3)
C17	0.041 (5)	0.013 (4)	0.028 (4)	−0.004 (3)	−0.004 (3)	−0.002 (3)
O1W	0.219 (12)	0.049 (5)	0.081 (7)	0.003 (6)	−0.018 (8)	−0.001 (5)
O2W	0.125 (7)	0.055 (5)	0.079 (6)	−0.016 (5)	−0.009 (5)	0.009 (4)
O3W	0.129 (17)	0.087 (13)	0.116 (16)	−0.012 (12)	0.015 (13)	0.014 (11)

### Geometric parameters (Å, º)

**Table:**

Cu1—Cu1^i^	2.6201 (18)	C6—C7	1.365 (11)
Cu1—O2	1.965 (5)	C7—H7	0.9500
Cu1—O3^i^	1.967 (5)	C8—C9	1.510 (9)
Cu1—O4^ii^	1.991 (5)	C9—H9A	0.9900
Cu1—O5^iii^	1.975 (5)	C9—H9B	0.9900
Cu1—N1	2.172 (6)	C9—C10	1.538 (9)
O1—C2	1.255 (9)	C10—H10A	0.9900
O2—C8	1.263 (9)	C10—H10B	0.9900
O3—C8	1.263 (8)	C10—C11	1.522 (10)
O4—C17	1.247 (8)	C11—C12	1.383 (10)
O5—C17	1.278 (8)	C11—C16	1.405 (10)
N1—C4	1.325 (9)	C12—H12	0.9500
N1—C5	1.332 (8)	C12—C13	1.366 (11)
N2—H2	0.87 (2)	C13—H13	0.9500
N2—C1	1.460 (10)	C13—C14	1.424 (12)
N2—C2	1.299 (10)	C14—H14	0.9500
C1—C1^iv^	1.480 (16)	C14—C15	1.376 (11)
C1—H1A	0.9900	C15—H15	0.9500
C1—H1B	0.9900	C15—C16	1.393 (10)
C2—C3	1.479 (11)	C16—C17	1.505 (10)
C3—C4	1.387 (10)	O1W—H1WA	0.85 (2)
C3—C7	1.400 (11)	O1W—H1WB	0.85 (2)
C4—H4	0.9500	O2W—H2WA	0.846 (18)
C5—H5	0.9500	O2W—H2WB	0.85 (2)
C5—C6	1.379 (10)	O3W—H3WA	0.84 (2)
C6—H6	0.9500	O3W—H3WB	0.84 (2)
			
O2—Cu1—Cu1^i^	81.66 (15)	C7—C6—C5	117.3 (7)
O2—Cu1—O3^i^	168.9 (2)	C7—C6—H6	121.3
O2—Cu1—O4^ii^	90.1 (2)	C3—C7—H7	119.5
O2—Cu1—O5^iii^	91.5 (2)	C6—C7—C3	121.1 (7)
O2—Cu1—N1	93.6 (2)	C6—C7—H7	119.5
O3^i^—Cu1—Cu1^i^	87.24 (15)	O2—C8—O3	125.1 (7)
O3^i^—Cu1—O4^ii^	89.0 (2)	O2—C8—C9	117.5 (6)
O3^i^—Cu1—O5^iii^	87.1 (2)	O3—C8—C9	117.4 (6)
O3^i^—Cu1—N1	97.5 (2)	C8—C9—H9A	109.1
O4^ii^—Cu1—Cu1^i^	80.87 (16)	C8—C9—H9B	109.1
O4^ii^—Cu1—N1	87.0 (2)	C8—C9—C10	112.6 (6)
O5^iii^—Cu1—Cu1^i^	87.57 (15)	H9A—C9—H9B	107.8
O5^iii^—Cu1—O4^ii^	168.0 (2)	C10—C9—H9A	109.1
O5^iii^—Cu1—N1	104.8 (2)	C10—C9—H9B	109.1
N1—Cu1—Cu1^i^	166.94 (17)	C9—C10—H10A	108.2
C8—O2—Cu1	126.3 (5)	C9—C10—H10B	108.2
C8—O3—Cu1^i^	119.7 (5)	H10A—C10—H10B	107.3
C17—O4—Cu1^v^	127.8 (5)	C11—C10—C9	116.5 (6)
C17—O5—Cu1^vi^	120.0 (5)	C11—C10—H10A	108.2
C4—N1—Cu1	123.1 (5)	C11—C10—H10B	108.2
C4—N1—C5	116.6 (6)	C12—C11—C10	120.6 (6)
C5—N1—Cu1	118.2 (5)	C12—C11—C16	117.4 (7)
C1—N2—H2	111 (6)	C16—C11—C10	121.9 (6)
C2—N2—H2	122 (6)	C11—C12—H12	118.3
C2—N2—C1	122.7 (7)	C13—C12—C11	123.3 (7)
N2—C1—C1^iv^	111.9 (9)	C13—C12—H12	118.3
N2—C1—H1A	109.2	C12—C13—H13	120.6
N2—C1—H1B	109.2	C12—C13—C14	118.9 (8)
C1^iv^—C1—H1A	109.2	C14—C13—H13	120.6
C1^iv^—C1—H1B	109.2	C13—C14—H14	120.6
H1A—C1—H1B	107.9	C15—C14—C13	118.8 (8)
O1—C2—N2	122.4 (8)	C15—C14—H14	120.6
O1—C2—C3	118.7 (7)	C14—C15—H15	119.4
N2—C2—C3	119.0 (7)	C14—C15—C16	121.1 (8)
C4—C3—C2	124.7 (7)	C16—C15—H15	119.4
C4—C3—C7	115.5 (7)	C11—C16—C17	123.0 (7)
C7—C3—C2	119.8 (7)	C15—C16—C11	120.4 (7)
N1—C4—C3	125.2 (7)	C15—C16—C17	116.6 (7)
N1—C4—H4	117.4	O4—C17—O5	123.4 (7)
C3—C4—H4	117.4	O4—C17—C16	119.1 (6)
N1—C5—H5	117.9	O5—C17—C16	117.4 (7)
N1—C5—C6	124.3 (7)	H1WA—O1W—H1WB	107 (3)
C6—C5—H5	117.9	H2WA—O2W—H2WB	106 (3)
C5—C6—H6	121.3	H3WA—O3W—H3WB	108 (4)
			
Cu1—O2—C8—O3	−3.5 (11)	C4—C3—C7—C6	−1.4 (12)
Cu1—O2—C8—C9	178.6 (5)	C5—N1—C4—C3	0.2 (11)
Cu1^i^—O3—C8—O2	2.1 (10)	C5—C6—C7—C3	1.4 (12)
Cu1^i^—O3—C8—C9	179.9 (5)	C7—C3—C4—N1	0.6 (11)
Cu1^v^—O4—C17—O5	−6.6 (11)	C8—C9—C10—C11	−72.9 (8)
Cu1^v^—O4—C17—C16	175.9 (5)	C9—C10—C11—C12	−1.4 (10)
Cu1^vi^—O5—C17—O4	1.6 (10)	C9—C10—C11—C16	179.9 (6)
Cu1^vi^—O5—C17—C16	179.1 (5)	C10—C11—C12—C13	−178.1 (7)
Cu1—N1—C4—C3	−163.0 (6)	C10—C11—C16—C15	175.8 (7)
Cu1—N1—C5—C6	163.9 (6)	C10—C11—C16—C17	−3.5 (11)
O1—C2—C3—C4	179.3 (8)	C11—C12—C13—C14	1.8 (13)
O1—C2—C3—C7	−0.3 (12)	C11—C16—C17—O4	−28.2 (11)
O2—C8—C9—C10	−40.7 (10)	C11—C16—C17—O5	154.2 (7)
O3—C8—C9—C10	141.3 (7)	C12—C11—C16—C15	−3.0 (11)
N1—C5—C6—C7	−0.7 (11)	C12—C11—C16—C17	177.7 (7)
N2—C2—C3—C4	−0.1 (12)	C12—C13—C14—C15	−2.0 (13)
N2—C2—C3—C7	−179.7 (7)	C13—C14—C15—C16	−0.2 (12)
C1—N2—C2—O1	−1.8 (13)	C14—C15—C16—C11	2.7 (12)
C1—N2—C2—C3	177.7 (7)	C14—C15—C16—C17	−177.9 (7)
C2—N2—C1—C1^iv^	−80.0 (12)	C15—C16—C17—O4	152.4 (7)
C2—C3—C4—N1	−179.0 (7)	C15—C16—C17—O5	−25.2 (10)
C2—C3—C7—C6	178.3 (7)	C16—C11—C12—C13	0.7 (11)
C4—N1—C5—C6	−0.1 (10)		

Symmetry codes: (i) −*x*+1/2, −*y*+1/2, −*z*+1; (ii) *x*, −*y*+1, *z*+1/2; (iii) −*x*+1/2, *y*−1/2, −*z*+1/2; (iv) −*x*+1, −*y*+1, −*z*+2; (v) *x*, −*y*+1, *z*−1/2; (vi) −*x*+1/2, *y*+1/2, −*z*+1/2.

### Hydrogen-bond geometry (Å, º)

**Table:**

*D*—H···*A*	*D*—H	H···*A*	*D*···*A*	*D*—H···*A*
N2—H2···O1*W*	0.87 (2)	2.03 (4)	2.844 (10)	155 (8)
C5—H5···O2	0.95	2.50	3.099 (9)	121
C6—H6···O3^vi^	0.95	2.48	3.332 (9)	149
O1*W*—H1*WA*···O2*W*	0.85 (2)	2.00 (11)	2.698 (13)	139 (15)
O1*W*—H1*WB*···O3*W*	0.85 (2)	2.10 (2)	2.79 (2)	139 (5)
O2*W*—H2*WA*···O5^iii^	0.85 (2)	2.09 (2)	2.909 (10)	162 (7)
O2*W*—H2*WB*···O1^v^	0.85 (2)	2.06 (2)	2.816 (9)	147 (5)
O3*W*—H3*WA*···O1^ii^	0.84 (2)	2.10 (2)	2.917 (19)	162 (9)
O3*W*—H3*WB*···O1^iv^	0.84 (2)	2.10 (2)	2.848 (19)	147 (6)

Symmetry codes: (ii) *x*, −*y*+1, *z*+1/2; (iii) −*x*+1/2, *y*−1/2, −*z*+1/2; (iv) −*x*+1, −*y*+1, −*z*+2; (v) *x*, −*y*+1, *z*−1/2; (vi) −*x*+1/2, *y*+1/2, −*z*+1/2.
